# The optimal period for oocyte retrieval after the administration of recombinant human chorionic gonadotropin in in vitro fertilization

**DOI:** 10.1186/s12884-022-04412-9

**Published:** 2022-03-07

**Authors:** Mhd Jawad Al Rahwanji, Homam Abouras, Mhd Said Shammout, Ray Altalla, Reem Al Sakaan, Nawras Alhalabi, Marwan Alhalabi

**Affiliations:** 1 Department of Artificial Intelligence, Faculty of Information Technology Engineering, Damascus University, Damascus, Syria; 2 Faculty of Medicine, Damascus University, Damascus, Syria; 3 Faculty of Medicine, Syrian Private University, Damascus, Syria; 4 Division of Reproductive Medicine, Embryology and Genetics, Faculty of Medicine, Damascus University, Damascus, Syria; 5 Assisted Reproductive Unit, Orient Hospital, Damascus, Syria

**Keywords:** IVF, Oocyte retrieval, IVF outcome, rhCG, Long GnRH agonist protocol

## Abstract

**Background:**

Our objective was to investigate the existence of an optimal period for oocyte retrieval in regards to the clinical pregnancy occurrence after the administration of recombinant human chorionic gonadotropin (rhCG) (Ovitrelle®).

**Methods:**

We studied the digital records of 3362 middle eastern couples who underwent in vitro fertilization (IVF) treatment between 2019 and 2021.

**Results:**

Through statistical testing, we found that there is a significant positive correlation between the oocyte retrieval period and the clinical pregnancy occurrence up to the 37th hour, where retrieval at the 37th hour was found to provide the most optimal outcome, especially in the case of gonadotropin-releasing hormone agonist (GnRHa) long protocol.

**Conclusions:**

This cohort study recommends retrieval at hour 37 after ovulation triggering under the described conditions.

## Background

Infertility affects millions of individuals of reproductive age across the world and poses a complex medical challenge with a wide range of implications spanning from legal, moral, and ethical to financial factors that impact the infertile couple and the society as a whole. Estimates show that around 48 million couples or 186 million individuals suffer from infertility worldwide [1].

IVF represents the solution to infertility, in cases where conventional medical treatment fails to alleviate the problem, where an egg is fertilized with sperm outside of the female reproductive system.

In IVF, ovulation is triggered by giving an injection of recombinant or nonrecombinant hCG then oocytes are retrieved, with retrieval taking place sometime between the 35th and the 38th hour after triggering at 1-h intervals. Next, insemination is conducted regardless of the method used through either maturated oocyte exposure to sperm or intra-cytoplasmic sperm injection (ICSI). Finally, clinical pregnancy occurrence marks the end of treatment [2].

To the best of our knowledge, there was no prior study of this scale in the medical literature to come out of the middle eastern region regarding the egg retrieval period or its relationship with clinical pregnancy occurrence. It is uncertain among gynecologists as to which hour is best for egg retrieval. Farrag et al. thoroughly tested the effect of using rhCG and found it to increase the rate of mature oocytes [3]. Conversely, we set out to measure the clinical pregnancy occurrence while taking into consideration the count of matured oocytes, fertilized eggs, embryos and high quality embryos on a large sample of middle eastern couples.

## Methods

### Data collection

We studied the data of all IVF patients at Orient Hospital between January 2019 and January 2021. The ethical approval was obtained from the ethical research committee at the Faculty of Medicine, Damascus University, with the approval of the Orient Hospital board of directors.

The patients’ digital records were reviewed retrospectively and data regarding patients’ medical history, history of IVF, hour of egg retrieval (ERH), eggs in metaphase II (M II), fertilized eggs (FE), embryo count, high quality embryo count, maturation rate (MR), fertilization rate (FR), cleavage rate (CR), high quality embryo rate (HQER), IVF protocol and clinical pregnancy after embryo transfer were collected. Patients’ history of IVF was incomplete as the number of cycles and their success rate were available but retrieval hours and other specific details in those cycles were not.

### Data revision

Our patients had an average age of 32.81 ± 6.38 years, shown as mean ± standard deviation (SD). Patients had BMIs mostly within normal ranges with no documented extreme cases and the patients were mainly of middle eastern origin. After the examination of the digital records, we excluded patients that had their IVF medically terminated, for example, due to OHSS or uterine bleeding or logistically terminated, which was due to the rough conditions the country has been through in the past few years which made it difficult to navigate within the country as well as any financial problems couples may have faced. All patients with a valid maturation rate, fertilization rate, cleavage rate, high quality embryo rate and clinical pregnancy occurrence were included. PCOS patients weren’t excluded. All patients were treated and had their TSH levels stabilized (between 0.27–2.5 μIU/ml) before undergoing the IVF treatment. A total of 3362 patients met the specified criteria.

### IVF protocol

All women who had IVF treatment were subjected to either long gonadotropin-releasing hormone (GnRH) agonist or GnRH antagonist protocol for pituitary suppression as mentioned in previous literature [4]. Other controlled ovarian hyperstimulation (COH) protocols weren’t used due to hospital policy because of the higher success rates achieved by the long and short protocols as reported in [5, 6]. For follicular growth stimulation, the patients received human menopausal gonadotropin (HMG) or recombinant follicle-stimulating hormone (rFSH), or both.

After ultrasound documentation of follicular maturity and testing of blood estradiol, 250 mcg (equal to 6500 IU) of recombinant human chorionic gonadotropin (rhCG) (Ovitrelle®, Merck Serono S.p.A, Modugno (BA), Italy) was administered at the time when three leading follicles reached 17–18 mm.

Following 35 to 38 h, we performed transvaginal ultrasound-guided oocyte retrieval. Which was done at 1 h intervals based on the time of arrival of the patient and their responsible doctor/biologist to the lab as well as the availability of the retrieval chambers. As such, the time of retrieval wasn’t scheduled based on how many eggs were to be expected. For fertilization of the matured oocytes, intracytoplasmic sperm injection (ICSI) was done. Three embryos were transferred to the uterine cavity with transabdominal ultrasound guidance in the cleavage stage (day 3). In some cases (1%), only two were transferred instead as per patient request. Eventually, the clinical pregnancy was deemed positive by both indicative hCG tests and ultrasonography evidence of a gestational sac in the uterine cavity.

### Statistical analysis

Statistical analysis was done using Pandas for the reading and manipulation of the spreadsheet, Matplotlib [7] for graph visualizations and Pandas [8, 9] for table creation, NumPy [10] for basic matrix calculations, SciPy [11] and Pingouin [12] which contains implementations for all the statistical tests and algorithms used. All the aforementioned are libraries written for the Python programming language.

The Kruskal-Wallis and the analysis of variance (ANOVA) tests were used. In addition, the Shapiro-Wilks normality test was used to confirm non-normality with significant *p*-values.

Nevertheless, we used both tests to boost our confidence in our results. Through the examination of box and whiskers plots, we found that the data of the groups in question followed an exponential distribution. Due to the large sample size, the use of ANOVA was justified, for it is robust enough to withstand non − normal data if the sample is large. The results of both tests were effectively identical. Pairwise t-tests and Mann Whitney U tests were used to investigate any significance. Analysis was performed as was advised by Petrie & Sabin (2019) [13]. A *p*-value less than 0.005 was required for an observation to be considered statistically significant as recommended by Benjamin et al. (2018) and Di Leo & Sardanelli (2020) [14, 15].

## Results

Between January 2019 and January 2021, a total of 3362 patients attended the Orient Hospital for IVF and met the inclusion criteria of the study. The patients varied by their egg retrieval hour (between 35 and 38).

**Fig. 1:**
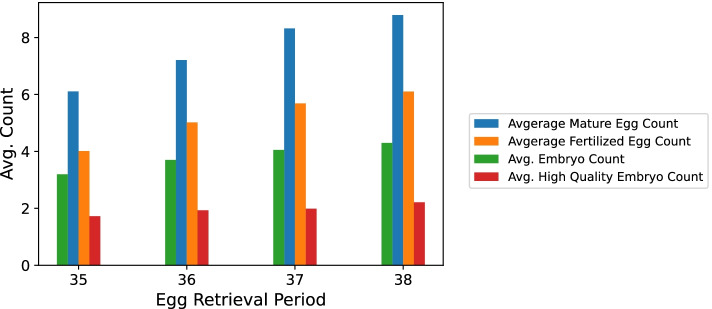
Comparing M II, FE, embryo count and HQ embryo count per ERH. Note. The positive trend in all variables except the HQ embryo count

**Fig. 2:**
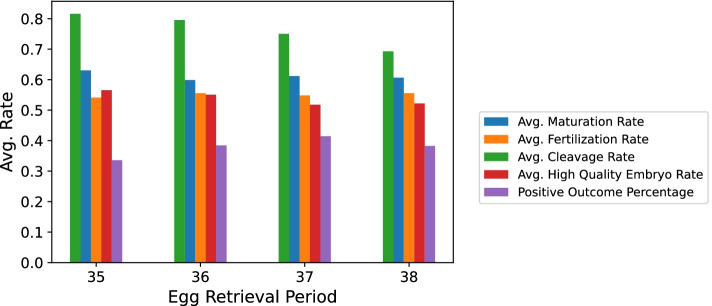
Comparing MR, FR, CR, HQER, and POP per ERH. *Note.* The orange and red bars have comparable heights across hours. The blue and red bars are inconsistent with the trend that the purple bars follow. The green bars are decreasing as the hours increase

We found that the group of hour 37 was significantly and consistently superior to the rest of the groups of ERHs except for the group of hour 38 (*p*-value > 0.005) as can be seen in Tables 1, 2 & 3 and Figs. 1 and 2. We also noticed a positive trend, where the group of hour 36 had significantly more eggs in metaphase II than the group of hour 35. Also, the group of hour 37 was better in terms of M II, FE, embryo count, HQ embryo count and clinical pregnancy positive occurrence percentage than both the group of hour 35 and the group of hour 36 but not the group of hour 38.

**Table 1:** Numerical values for Figs. 1 and 2

ERH	Sample Size	Avg. M II ± SD	Avg. MR ± SD	Avg. FE ± SD	Avg. FR ± SD	Avg. Embryo ± SD	Avg. CR ± SD	Avg. HQ Embryo ± SD	Avg. HQER ± SD	Outcome
35	509	6.1 ± 4.8	.63 ± .21	4.0 ± 3.5	.54 ± .28	3.2 ± 1.9	.82 ± .3	1.7 ± 1.4	.57 ± .31	.33
36	1653	7.2 ± 5.0	.60 ± .19	5.0 ± 3.7	.56 ± .24	3.7 ± 1.9	.8 ± .29	1.9 ± 1.4	.55 ± .29	.38
37	1072	8.3 ± 5.5	.61 ± .18	5.7 ± 4.1	.55 ± .24	4.1 ± 1.9	.75 ± .3	2.0 ± 1.5	.52 ± .29	.41
38	128	8.8 ± 5.1	.61 ± .18	6.1 ± 4.0	.56 ± .25	4.3 ± 1.9	.69 ± .34	2.2 ± 1.6	.52 ± .31	.38

Note. Uneven distribution of the samples among the groups of ERHs. *FR* fertilization rate, *MR* maturation rate, *SD* Standard Deviation, Outcome = Clinical Pregnancy Occurrence Positive Percentage, *CR* Cleavage Rate, *HQER* High Quality Embryo Rate

**Table 2:** Comparing ERHs using one-way ANOVA based on M II, FE, embryo count and HQ embryo count

IV	DV	SS	DF	MS	p-value
Egg Retrieval Period	M II	2051.4	3	683.8	*p* < .001
	Fertilized egg count	1109.4	3	369.8	*p* < .001
	Embryo count	296.9	3	99.0	*p* < .001
	HQ embryo count	35.2	3	11.7	*p* < .001

*IV* Independent Variable, *DV* Dependent Variable, *DF* Degrees of Freedom, *SS* Square of Sums, *MS* Mean Squares, *HQ* High Quality

**Table 3:** ANOVA post-hoc with pairwise t-tests based on M II, FE, embryo count and HQ embryo count

**DV is M II**							
**A**	**B**	**A**		**B**		**dof**	**p-value**
		**M**	**SD**	**M**	**SD**		
35	36	6.1	4.8	7.2	5.0	868.2	*p* < .001
35	37	6.1	4.8	8.3	5.5	1125.6	*p* < .001
35	38	6.1	4.8	8.8	5.1	188.0	*p* < .001
36	37	7.2	5.0	8.3	5.5	2124.5	*p* < .001
36	38	7.2	5.0	8.8	5.1	146.4	*p* < .001
37	38	8.3	5.5	8.8	5.1	164.2	NS
**DV is FE**							
**A**	**B**	**A**		**B**		**dof**	**p-value**
		**M**	**SD**	**M**	**SD**		
35	36	4.0	3.5	5.0	3.7	901.4	*p* < .001
35	37	4.0	3.5	5.7	4.1	1168.5	*p* < .001
35	38	4.0	3.5	6.1	4.1	176.1	*p* < .001
36	37	5.0	3.7	5.7	4.1	2127.0	*p* < .001
36	38	5.0	3.7	6.1	4.1	144.1	.004
37	38	5.7	4.1	6.1	4.1	159.7	NS
**DV is Embryo count**							
**A**	**B**	**A**		**B**		**dof**	**p-value**
		**M**	**SD**	**M**	**SD**		
35	36	3.2	1.9	3.7	1.9	836.4	*p* < .001
35	37	3.2	1.9	4.0	2.0	1027.1	*p* < .001
35	38	3.2	1.9	4.3	1.9	193.7	*p* < .001
36	37	3.7	1.9	4.0	2.0	2216.2	*p* < .001
36	38	3.7	1.9	4.3	1.9	146.3	.0049
37	38	4.0	2.0	4.3	1.9	160	NS
**DV is HQ Embryo count**							
**A**	**B**	**A**		**B**		**dof**	**p-value**
		**M**	**SD**	**M**	**SD**		
35	36	1.7	1.4	1.9	1.4	872.7	NS
35	37	1.7	1.4	2.0	1.5	1063.1	.003
35	38	1.7	1.4	2.2	1.6	174.3	NS
36	37	1.9	1.4	2.0	1.5	2237.1	NS
36	38	1.9	1.4	2.2	1.6	142.3	NS
37	38	2.0	1.5	2.2	1.6	152.4	NS

Note. When conducting pairwise t-tests independent variables (IVs), in this case ERH groups, are compared in a pairwise manner, where each group is compared once with each of the rest, using the t-test. As a result, A represents one of the ERH groups and B represents another. Results remained consistent for all DVs. The group of hour 38 had the smallest sample size of 128, so its results may not be as reliable as other groups of ERHs but statistically sound and acceptable, nonetheless. *NS* not significant, *SD* standard deviation, *DV* Dependent Variable, *dof* Degrees of Freedom, *SD* Standard Deviation, *M* Mean, *HQ* High Quality

We plotted ERH against the clinical pregnancy positive occurrence percentage (POP). Results in order were from most favorable outcome to least: 37 > 36 > 38 > 35. The perceived positive trend leading up to the group of hour 37 is shown in Table 1 and Fig. 2.

We divided our sample into groups of patients who had their IVF using long gonadotropin-releasing hormone (GnRH) agonist (Group L), or GnRH antagonist (Group A) for pituitary suppression for further investigation in accordance with the methodology of Depalo et al. (2009) [4]. A total of 2838 patients had IVF using the L protocol (L Group) whereas a total of 446 patients had an A protocol IVF (A Group). A statistical significance was found between distinct hours of retrieval for patients in the L Group, both when we had M II, FE and embryo count as the dependent variable, whereas patients in the A Group showed no statistical significance, as shown in Tables 4 & 5.

**Table 4:** Comparing ERH using ANOVA based on M II, FE, embryo count, HQ embryo count for L and A Groups

IV	DV	Protocol	SS	DF	MS	***p***-value
Egg Retrieval Period	M II	L	628.1	3	209.4	*p* < .001
		A	30.7	3	10.2	NS
	Fertilized	L	392.2	3	130.7	*p* < .001
		A	17.2	3	5.7	NS
	Embryo count	L	101.3	3	33.8	*p* < .001
		A	13.8	3	4.6	NS
	HQ Embryo count	L	9.1	3	3.0	NS
		A	2.1	3	0.7	NS

*IV* Independent Variable, *DV* Dependent Variable, *DF* Degrees of Freedom, *SS* Square of Sums, *MS* Mean Squares, *HQ* High Quality

**Table 5:** ANOVA post-hoc with pairwise t-tests based on M II, FE and embryo count for L Group

**DV is M II**							
**A**	**B**	**A**		**B**		**dof**	***p*-value**
		**M**	**SD**	**M**	**SD**		
35	36	7.3	4.9	7.7	5.0	470.3	NS
35	37	7.3	4.9	8.5	5.5	579.7	*p* < .001
35	38	7.3	4.9	8.9	5.1	219.8	.003
36	37	7.7	5.0	8.5	5.5	2031.6	*p* < .001
36	38	7.7	5.0	8.9	5.1	144.8	NS
37	38	8.5	5.5	8.9	5.1	160.7	NS
**DV is FE**							
**A**	**B**	**A**		**B**		**dof**	**p-value**
		**M**	**SD**	**M**	**SD**		
35	36	4.7	3.6	5.3	3.8	482.0	NS
35	37	4.7	3.6	5.8	4.1	588.9	*p* < .001
35	38	4.7	3.6	6.2	4.1	205.3	*p* < .001
36	37	5.3	3.8	5.8	4.1	2055.7	.004
36	38	5.3	3.8	6.2	4.1	142.8	NS
37	38	5.8	4.1	6.2	4.1	156.0	NS
**DV is Embryo count**							
**A**	**B**	**A**		**B**		**dof**	**p-value**
		**M**	**SD**	**M**	**SD**		
35	36	3.6	1.9	3.9	1.9	459.2	NS
35	37	3.6	1.9	4.1	1.9	533.7	*p* < .001
35	38	3.6	1.9	4.4	1.9	226.4	.001
36	37	3.9	1.9	4.1	1.9	2109.9	.005
36	38	3.9	1.9	4.4	1.9	144.7	NS
37	38	4.1	1.9	4.4	1.9	156.3	NS

Note. When conducting pairwise t-tests independent variables (IVs), in this case ERH groups, are compared in a pairwise manner, where each group is compared once with each of the rest, using the t-test. As a result, A represents one of the ERH groups and B represents another. *DV* Dependent Variable, *dof* Degrees of Freedom, *SD* Standard Deviation, *M* Mean

**Fig. 3:**
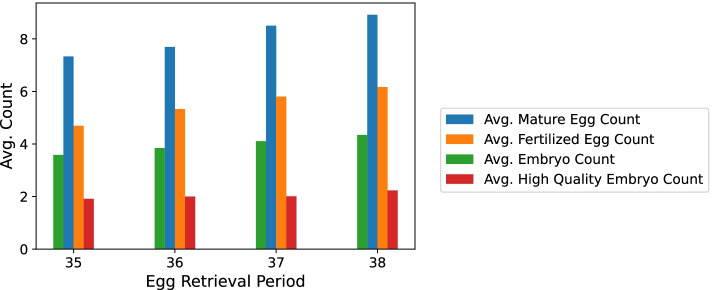
M II, FE, embryo count and HQ embryo rate per ERH (L Group). Note. The positive trend is in line with Fig. 1 for all variables except HQ embryo rate

We perceived the same positive trend in M II, FE, embryo count and clinical pregnancy occurrence in the L Group as the one we found in the sample overall, as can be seen in Table 6 and Fig. 3.

**Table 6:** Summary of the statistics done per ERH for the L group. (Used to plot Figs. 4 and 3)

ERH	Sample Size	Avg. M II ± SD	Avg. MR ± SD	Avg. FE ± SD	Avg. FR ± SD	Avg. Embryo ± SD	Avg. CR ± SD	Avg. HQ Embryo ± SD	Avg. HQER ± SD	Outcome
35	316	7.3 ± 5.0	.62 ± .19	4.7 ± 3.7	.52 ± .26	3.2 ± 1.9	.79 ± .31	1.9 ± 1.4	.56 ± .3	.39
36	1391	7.7 ± 5.0	.60 ± .19	5.3 ± 3.8	.54 ± .23	3.7 ± 1.9	.78 ± .29	2.0 ± 1.4	.54 ± .28	.40
37	1007	8.5 ± 5.5	.61 ± .18	5.8 ± 4.1	.55 ± .24	4.1 ± 1.9	.74 ± .31	2.0 ± 1.5	.51 ± .29	.42
38	124	8.9 ± 5.1	.61 ± .17	6.2 ± 4.1	.55 ± .25	4.3 ± 1.9	.69 ± .34	2.2 ± 1.7	.52 ± .31	.40

Note. Distribution of the groups of ERHs in L Group. The mean of oocytes in metaphase 2, maturation rate, fertilized eggs, fertilization rate. *SD* Standard Deviation. Outcome = Clinical Pregnancy Occurrence Positive Percentage

We plotted the average oocyte count for each of the ERH groups in Fig. 5 and noticed that more eggs were retrieved the later the ERH. We also viewed the distribution of patients who were treated using the short protocol (A Group) across ERHs as is shown in the histogram in Fig. 6. Lastly, the mean oocyte count per ERH for both groups (A Group and L Group) was plotted. One can see when looking at them side-by-side in Fig. 7 how the ranges of average oocyte count differs between the two groups.

## Discussion

We found a significant positive trend in our sample among the groups of ERHs based on M II, FE, embryo count and clinical pregnancy occurrence up to hour 37 where the trend ceased to exist between hour 37 and 38. This may be due to either the latter’s relatively small sample or that at hour 38 the eggs might have grown too old to perform well.

As for high quality embryo count we failed to find a significance. In order to explain why, we should first explain what was done in detail. We observed the oocytes’ development at four phases: oocyte maturation, oocyte fertilization, embryo formation and high quality embryo formation. At each phase we measured the mean oocyte count for each ERH. Then, we studied the correlation between those means for each phase. The later the phase, the weaker the correlations between means became. That can be explained by the fact that less and less oocytes/embryos make it from one phase to the next. Since counting high quality embryos happens late in the IVF cycle, we failed to find the same trend in the HQ embryo formation phase as we did in the first three phases.

We showed that hour 37 significantly had the highest average M II and significantly had the highest outcomes in average FE, average embryo count and clinical pregnancy occurrences.

**Fig. 4:**
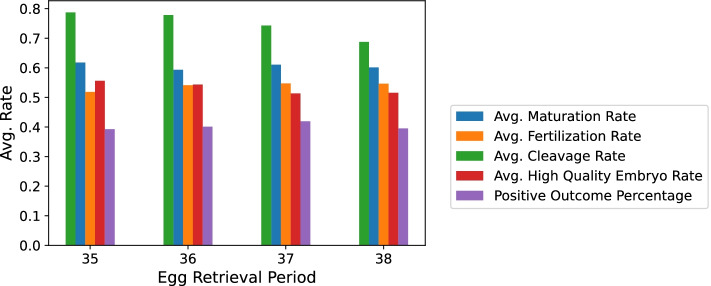
MR, FR, CR, HQER and POP per ERH (L Group). Note. The graph is in line with Fig. 2 in all 5 bar colors across the hours

**Fig. 5:**
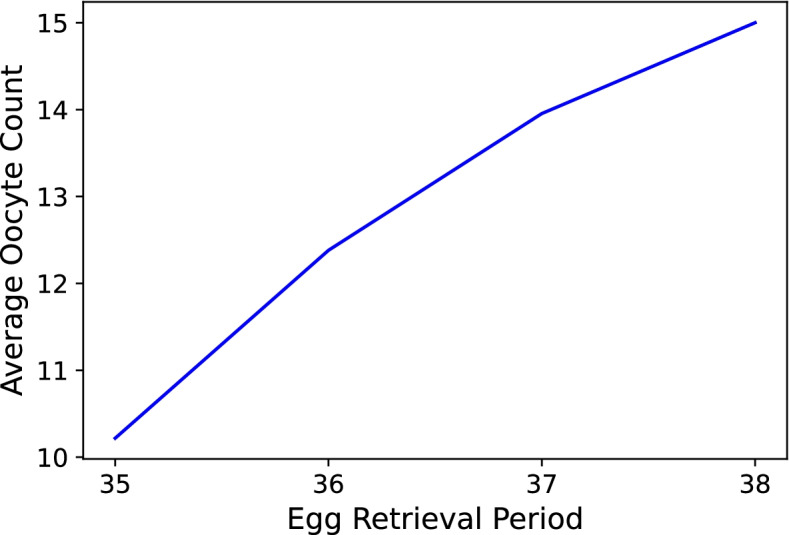
Average oocyte count per group of ERHs. Note. The rise between hours 35 and 36 is much greater than the rise between hours 36–37 and hours 37–38

With regards to the maturation rate, which is the percentage of how many eggs matured (M II) out of all retrieved oocytes, Figs. 2 and 4 show that the MR did not follow the discovered trend. We presume that the reason for that is that the earlier we retrieve, the fewer immature oocytes are released from their follicles. Therefore, we get an inherently higher MR at 35 which is why the mean MRs can be viewed as misleading. We confirmed this by examining the average oocyte count for each of the hours in Fig. 5.

The fertilization rate, which is the percentage of how many eggs got fertilized out of all mature eggs that had been injected with sperm in ICSI, was also noted to remain somewhat constant. That can simply be explained by the fact that we are dividing the increasing amount of FEs per hour by the increasing amount of M II per hour resulting in almost the same rates across the hours.

The cleavage rate is the percentage of fertilized eggs that made it into day-3 embryos. It seemed to follow a negative correlation with the ERHs. If we take a look at Fig. 1 we can see that the gap between embryo count and fertilized egg count increases along the ERH, meaning the positive trend in embryo count has an inferior slope when compared to that of the fertilized egg count. Hence, the negative trend perceived in the cleavage rate.

The high quality embryo rate (HQER) is defined as the total number of grade one embryos by the total number of embryos. The average HQER remained the same across all 4 h as was the case with the fertilization rate.

We divided our patients into those who had their IVF treatment using the L and A protocols. We perceived the same significant trend and the same best group of ERH in the L Group as well as in the sample overall, denoting that the 37th hour is the best hour for egg retrieval especially in the L Group who took the rHCG stimulation drug.

**Fig. 6:**
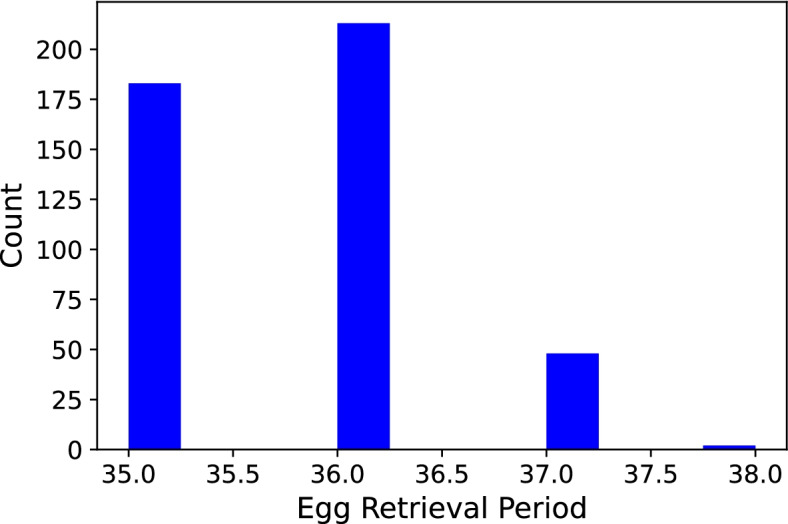
Histogram of ERHs (A Group). Note. The steep descent in the number of samples for hours 37 and 38

**Fig. 7:**
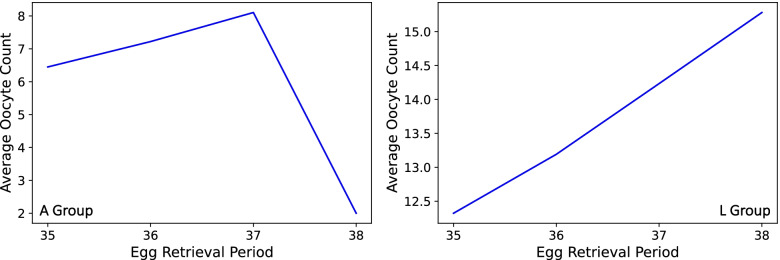
Average oocyte count per ERH (A Group and L Group). Note. The difference between the ranges of average oocyte count values (Y-axis)

On the other hand, the A protocol group showed no significant trend whatsoever nor did we find a significantly best hour either. We describe a couple of observations, the first being that the overall sample is smaller than that of the L protocol. Also, the share of patients of hours 37 and 38 in our sample is very small when compared to that of hours 35 and 36 as shown in Fig. 6. This is to be expected in the A protocol [4]. Furthermore, the average count of oocytes is much less in the A Group than in the L Group as can be seen when comparing graphs in Fig. 7.

Arieh Raziel et al. (2006) analyzed a select group of poor responders. 15% of our sample were considered poor responders and were treated using short protocol (Group A). As such, we were unable to meaningfully compare with their report [16]. On the other hand, our findings echoed those of Xi Shen et al. (2020) and Chun-I Lee et al. (2020) where they too perceived a positive correlation and consequently better results were found the longer the patients were exposed to hCG [17, 18]. Chun-I Lee et al. (2020) went beyond the 37th hour, investigating the correlation further [18]. Surprisingly, Julia K Bosdou et al. (2015) showed that there were no significant differences between 36 h and 38 h regardless of protocol. Moreover, they failed to perceive a positive correlation between the hours of retrieval [19]. All in all, our report remains unique as it demonstrates figures and results from a large sample of middle eastern patients. The scale of the study lends confidence in its results previously unknown to the region.

## Conclusion

We conducted a cohort study on a large sample of 3362 couples who visited Orient Hospital for IVF treatment. We sought to determine the best hour to retrieve oocytes of the hours 35, 36, 37, and 38. We presented statistical evidence to support our hypothesis, being that, firstly, the 37th hour after the administration of the rhCG (Ovitrelle®) trigger proved to be the best overall when using the long protocol for IVF in particular. That is when taking into consideration mature oocyte count, fertilized oocyte count, embryo count and clinical pregnancy occurrence.

We concluded our study with statistical concordance in our results even when groups were analyzed by protocols used. We highly recommend the use of rHCG for its benefits and to retrieve at hour 37 for patients treated using long protocol if applicable.

We were unable to determine how the 38th-hour factors in, so we hope to see further research in the form of controlled clinical trials to further evaluate the best possible methodology. Further work could be done to find the best retrieval hour and parameters for the antagonist IVF protocol.

## Data Availability

The dataset used in this research is available from the corresponding author on reasonable request. The python notebook containing the statistical tests and results of this research is available from the corresponding author on reasonable request.

## Abbreviations

rhCG: Recombinant human chorionic gonadotropin
IVF: In vitro fertilization
GnRH: Gonadotropin-releasing hormone
GnRHa: Gonadotropin-releasing hormone agonist
ICSI: Intracytoplasmic sperm injection
ERH: Hour of egg retrieval
M II: Eggs in metaphase II
FE: Fertilized eggs
MR: Maturation rate
FR: Fertilization rate
CR: Cleavage rate
HQER: High quality embryo rate
SD: Standard deviation
HMG: Human menopausal gonadotropin
rFSH: Recombinant follicle-stimulating hormone
ANOVA: Analysis of variance
POP: Positive occurrence percentage
